# Mimicking the Annulus Fibrosus Using Electrospun Polyester Blended Scaffolds

**DOI:** 10.3390/nano9040537

**Published:** 2019-04-03

**Authors:** Alyah H. Shamsah, Sarah H. Cartmell, Stephen M. Richardson, Lucy A. Bosworth

**Affiliations:** 1 School of Materials, Faculty of Science and Engineering, The University of Manchester, Oxford Road, Manchester M13 9PL, UK; alyahhhh.shamsah@postgrad.manchester.ac.uk (A.H.S.); sarah.cartmell@manchester.ac.uk (S.H.C.); 2 School of Biology, Faculty of Biology, Medicine and Health, The University of Manchester, Oxford Road, Manchester M13 9PL, UK; s.richardson@manchester.ac.uk; 3 Department of Eye and Vision Science, Institute of Ageing and Chronic Disease, 6 West Derby Street, University of Liverpool, Liverpool L7 8TX, UK

**Keywords:** electrospinning, intervertebral discs, annulus fibrosus, polycaprolactone, poly(L-lactic) acid

## Abstract

Treatments to alleviate chronic lower back pain, caused by intervertebral disc herniation as a consequence of degenerate annulus fibrosus (AF) tissue, fail to provide long-term relief and do not restore tissue structure or function. This study aims to mimic the architecture and mechanical environment of AF tissue using electrospun fiber scaffolds made from synthetic biopolymers-poly(ε-caprolactone) (PCL) and poly(L-lactic) acid (PLLA). Pure polymer and their blends (PCL%:PLLA%; 80:20, 50:50, and 20:80) are studied and material properties-fiber diameter, alignment, % crystallinity, tensile strength, and water contact angle-characterized. Tensile properties of fibers angled at 0°, 30°, and 60° (single layer scaffolds), and ±0°, ±30°, and ±60° (bilayer scaffolds) yield significant differences, with PCL being significantly stiffer with the addition of PLLA, and bilayer scaffolds considerably stronger. Findings suggest PCL:PLLA 50:50 fibers are similar to human AF properties. Furthermore, in vitro culture of AF cells on 50:50 fibers demonstrates attachment and proliferation over seven days. The optimal polymer composition for production of scaffolds that closely mimic AF tissue both structurally, mechanically, and which also support and guide favorable cell phenotype is identified. This study takes a step closer towards successful AF tissue engineering and a long-term treatment for sufferers of chronic back pain.

## 1. Introduction

Injury to, or degeneration of the annulus fibrosus (AF) can result in herniation of the intervertebral disc (IVD) and compression of the nerve root, resulting in chronic lower back pain [1]. Treatments such as medications or surgical procedures are able to relieve symptoms but fail to restore the native structure and function [1]. Regenerative medicine is a promising approach for the treatment of degenerative AF tissues [2]. However, the complex architectural and mechanical environment of the AF makes synthesis of a biomimetic artificial substrate challenging. The AF is composed of 15–25 highly oriented concentric lamellae that surround the nucleus pulposus (NP). Each lamella is oriented at 30° to adjacent layers and collagen fibers within each lamella are aligned parallel to each other [3,4]. This cross-aligned fibrous structure is critical for complex mechanical behavior, such as tension, which has nonlinear anisotropic behavior. In order to mimic the properties of native AF using biomaterials, it is essential the scaffold closely replicate the structural architecture and physical properties.

Electrospinning is a technique favored in the biomaterials and regenerative medicine field as it readily produces nanofiber substrates that mimic the architecture of extracellular matrix (ECM) to guide and stimulate desired cell morphology and phenotype [5]. Aligned electrospun nanofibers have previously been shown to successfully mimic native AF lamellae [6]. Polycaprolactone (PCL) electrospun fibers are widely studied for IVD tissue engineering due to their biocompatibility, long-term biodegradability and resemblance to native AF tissue [7]. However, PCL possesses low Young’s modulus, which could consequently result in functional failure of the AF, as the tissue needs to be sufficiently stiff to withstand high loads. To overcome this limitation, we propose a new composition of electrospun fibers based on a blend of PCL with poly-L-lactic acid (PLLA), a well-known biocompatible and rigid polymer, to ensure fibers continue to mimic the native AF, whilst also conferring desirable mechanical properties. 

Thus, we hypothesize that electrospun nanofibers made from synthetic, degradable polyesters will closely mimic the properties of the annulus fibrosus and could be used for its regeneration. To address this hypothesis, this study aims to determine the ideal blend of PCL with PLLA to closely replicate the morphological, structural, and mechanical properties of AF lamella sheets in order to take a step closer towards the repair of degenerate IVD [8]. 

## 2. Materials and Methods

### 2.1. Characterizing IVD and AF Tissue

#### 2.1.1. Lumbar Disc Dissection

Two lumbar IVDs were dissected from two 6-week postnatal female pigs (10–12 Kg) obtained from a local abattoir and processed within 3 h of slaughter for SEM imaging and histology study.

#### 2.1.2. Scanning Electron Microscopy (SEM)

AF tissue samples were dissected from whole discs (*n* = 2) using sterile disposable scalpels. Samples were fixed in 3% v/v glutaraldehyde in phosphate buffered saline (PBS) for 24 h then dehydrated in serial gradients of ethanol in deionized water (50, 70, 90, and 100% v/v) (2 × 3 min). Samples were chemically dried following repeated immersion in hexamethyldisilazane (5 min) and left to air-dry. Samples were mounted onto aluminum stubs with carbon tabs and gold coated (10 nm layer thickness) before imaging (Philips XL30 SEM, Edinburgh, United Kingdom). Images of collagen fibers within the outer AF were captured at high magnification (×6000 and diameters (*n* = 100) measured using Image J software (1.48v, LI-COR Technology, Nebraska, NE, USA). 

#### 2.1.3. Histology

Whole discs (*n* = 2) and AF tissue segments (*n* = 2) were fixed overnight in 10% neutral buffer formalin then processed for histology (Tissue-Tek VIP 2000) and subsequently embedded in paraffin wax and hardened. Tissues were sectioned (5 μm) using a microtome (Leica-RM2145, Leica Biosystem UK Ltd, UK), de-waxed in xylene and subsequently dehydrated in descending concentrations of ethanol in deionized water (100, 90, 70, and 50% v/v) to tap water (3 min). Tissues were stained with haematoxylin and eosin (H&E), Gomori trichrome and picrosirius red. Samples were imaged under light and polarized light using confocal microscopy (Leica SP8, ×75, Leica Biosystem UK Ltd, UK).

### 2.2. Mimicking the AF Tissue Using Electrospinning

#### Scaffold Fabrication

Polycaprolactone (PCL; inherent viscosity = 1.3 dL/g; Purac Biomaterials), and poly-L-lactic acid (PLLA; inherent viscosity = 3.2 dL/g; Purac Biomaterials), were dissolved at room temperature in 1,1,1,3,3,3-hexafluoro-2-propanol (HFIP; Sigma-Aldrich) to prepare 10% w/v and 5% w/v solutions, respectively. From these, three PCL-PLLA solutions were prepared to the following percentage ratios (PCL%:PLLA%) 80:20, 50:50, and 20:80. Aligned electrospun nanofibrous scaffolds were collected on a rotating mandrel using the following electrospinning parameters: Flow rate: 1 mL/h, tip-to-collector distance: 20 cm, applied voltage: 20 kV, and mandrel speed: 600 rpm.

### 2.3. Material Characterization of AF Electrospun Biomimics

#### 2.3.1. Scanning Electron Microscopy (SEM)

Electrospun fiber scaffolds were individually mounted on SEM stubs with carbon tabs and coated with platinum (10 nm layer thickness). Fiber morphology was evaluated using SEM (Hitachi S-3000N, Berkshire, United Kingdom). Fiber diameter (*n* = 170) was measured using high magnification images (×6000, EDAX-AMETEK Material Analysis Division, New-Jersey, USA ) and Image J software (1.48v, LI-COR Technology, Nebraska, USA). Fiber orientation (*n* = 100) was determined from lower magnification images (×1.8 k, EDAX-AMETEK Material Analysis Division, New-Jersey, USA) and Image J was used to measure the angle between each fiber relative to the mandrel’s axis of rotation.

#### 2.3.2. Differential Scanning Calorimetry (DSC)

Thermal properties of PCL, PLLA, and blended scaffolds (*n* = 3) were determined by DSC (Q20, TA Instruments) under nitrogen gas flow. Specimens were heated to 250 °C, quenched to −90 °C, then heated to 250 °C at a rate of 10 °C/min. Melting point (Tm) and Enthalpy of Fusion (ΔHf) were determined from the third heat cycle. Percentage crystallinity was determined by comparing Enthalpies of Fusion for PCL (139.5 J/g) and PLLA (93 J/g) at 100% crystallinity [9].

#### 2.3.3. Tensile Testing

**Figure 1:**
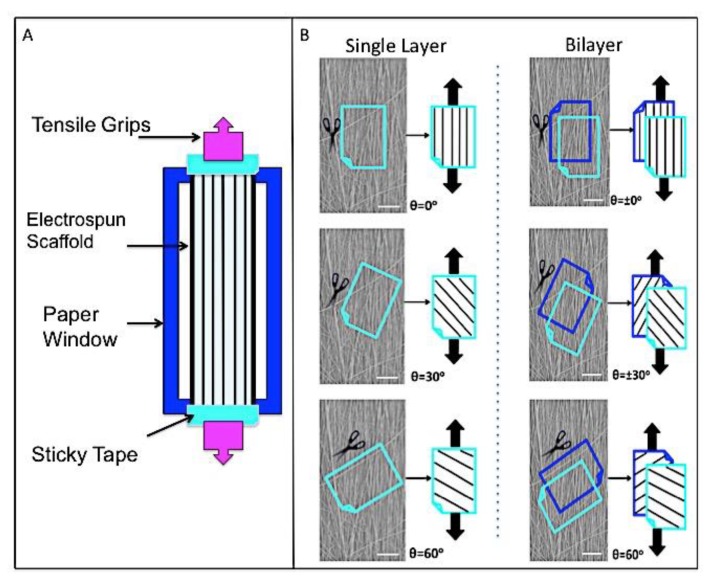
(**A**) Schematic presenting sample preparation for tensile testing. (**B**) Schematic on SEM images demonstrating position of samples cut to required angle relative to main fiber direction; and subsequent tensile test position for single and double layer (black arrows indicate direction of applied load).

Samples were prepared for tensile testing as outlined in Figure 1A. Briefly, electrospun samples (mean thickness 80 μm) were cut (22 × 5 mm) and mounted onto paper windows with sticky tape. Paper windows were gripped within tensile grips of an Instron (Model 1122), allowing a 20 mm gauge length, and the sides cut. Uniaxial tension was applied using a 10 N load cell and 0.1% strain rate. Testing was carried-out on single layer and bilayer samples (Figure 1B). For single layer, fibers were angled at 0°, 30°, and 60° relative to the direction of tensile load (*n* = 8). For opposing bilayer samples, fibers were angled at ±0°, ±30°, and ±60° relative to the direction of tensile load (*n* = 8). Stress-strain curves were obtained for each sample and Young’s modulus, ultimate tensile strength, and maximum strain percentage calculated. 

#### 2.3.4. Water Contact Angle (WCA)

The wettability of the electrospun scaffolds was assessed using Kruss system (DSA 100 goniometer) with static sessile drop. Samples were held horizontally on glass slides within the field of view, and an 8 μL droplet of distilled water released from an 8-gauge needle onto the scaffold surface. Sample wettability (*n* = 5) was tested before and after 12 h treatment in cell culture media (Dulbecco’s Modified Eagle’s medium, DMEM, Sigma-Aldrich) supplemented with 10% fetal bovine serum (FBS; Gibco). Before testing, samples were vacuum-dried at room temperature for 4 h. 

### 2.4. In Vitro Cell Response to AF Electrospun Biomimics

#### 2.4.1. Cell Culture

Due to a lack of healthy, primary human AF cells being available, animal-derived (Bovine) AF cells were obtained from the cell bank at the Division of Cell Matrix Biology and Regenerative Medicine, The University of Manchester, UK). AF cells were cultured to passage 3 at 37 °C and 5% CO_2_ in 75 cm^2^ sterile flasks with Dulbecco’s Modified Eagle’s Medium (DMEM) containing 4.5 g/L glucose, 5% sodium pyruvate 10% FBS, 1% antibiotic, and 50 g/mL ascorbic acid (Gibco). PCL/PLLA (50:50) blend scaffolds were individually held within 12-well CellCrown inserts (Sigma-Aldrich) and placed into 12-well plates (ThermoFisher). Samples were disinfected in 70% v/v ethanol in distilled water and pre-wetted for 12 h in culture media. This media was removed and 200 μL of AF cell suspension (1 × 10^5^ cells/sample) was evenly distributed over the surface of each scaffold. Samples were left undisturbed in the incubator (Jencons-PLS, Bedfordshire, United Kingdom) for 30 min to allow initial cell attachment before 2 mL medium was added to each well. Cell-seeded scaffolds were cultured for one week with media changes every second day.

#### 2.4.2. Cell Metabolic Activity

Alamar blue was used to measure the metabolic activity of AF cells at 1 and 7 days on the PCL/PLLA 50:50 scaffolds (*n* = 3). Resazurin salt (Sigma) was dissolved in PBS at a concentration of 0.125 mg/mL and filter-sterilized to create a stock Alamar blue solution. A 1:9 dilution of stock solution was added to fresh media in each well and incubated for 4 h. For each sample, 200 μL was transferred (in triplicate) to a 96-well plate and read by a fluorescent plate reader (Biotek) at 530 nm excitation and 590 nm emission.

#### 2.4.3. Cell Morphology

Cell morphology was assessed at 1 and 7 days using SEM and confocal microscopy. For SEM (Hitachi S3000N VPSEM), samples (*n* = 2) were washed in PBS and fixed in 2.5% v/v glutaraldehyde in PBS at 4 °C for 2 h. As previously described, samples were dehydrated through increasing concentrations of ethanol in distilled water (50%–100% v/v), chemically dried in hexamethyldisilazane, mounted on carbon-tabbed stubs, and gold-sputter coated. For confocal microscopy (Leica SP8), samples were fixed with 10% neutral buffered formalin for 30 min, washed with PBS, permeabilized for 1 h with 0.1% Triton X-100 in PBS (Sigma) then blocked with 2% bovine serum albumin (Sigma-Aldrich) at 37 °C for 1 h. F-actin stain (Alexa-Fluor488 phalloidin 1:300, Life Technologies) was applied and incubated for a further 2 h at 37 °C. Samples were subsequently washed with PBS and placed on glass coverslips for imaging.

### 2.5. Statistical Analysis

All quantitative data were statistically analyzed using Graphpad Prism software (v7) and checked for normality. Normally distributed data is presented as mean ± standard deviation and not normally distributed presented as box and whisker plots. Statistical analyses were performed using either unpaired t-test with Welch’s correction or two-way ANOVA, followed by Kruskal-Wallis with Dunn’s multiple comparison test, Bonferroni post-tests, or multiple comparison Tukey’s tests. *p* < 0.05 was considered significant.

## 3. Results

### 3.1. Characterizing the IVD and AF Tissue

**Figure 2:**
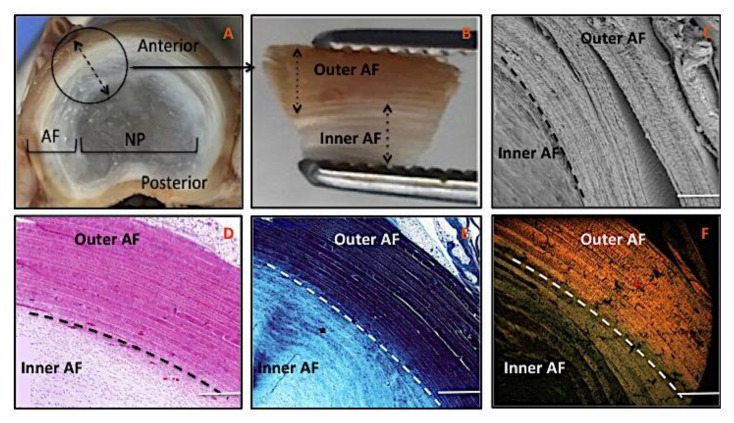
(**A**): Photograph of a dissected porcine lumbar intervertebral disc. (**B**): Outer and inner compartments of porcine AF tissue (**C**): SEM image demonstrating outer and inner regions of the AF tissue at high magnification. (**D**): H&E histology stain of AF tissue highlighting parallel lamellae. (**E**): Gomori Trichrome histology stain highlighting collagen in blue. (**F**): Picrosirius red histology stain viewed under polarized light. (Scale bar, C-F = 500 μm).

The gross anatomical structure of IVD clearly demonstrates its biphasic structure, with outer-fibrous AF tissue enclosing the gel-like, soft tissue of the nucleus pulposus (NP) (Figure 2A). Closer examination of AF tissues highlighted a demarcation between outer and inner AF lamellae (Figure 2B). Scanning Electron Microscopy (SEM) and histology images of transverse tissue sections further exhibited this distinction with outer AF lamellae appearing tightly packed with little visible spacing between lamella compared to inner lamellae (Figure 2C,D). Gamori stain showed both outer and inner AF tissue regions were collagen-rich, but staining was less dense in the inner region, which further demonstrated the concentration and parallel arrangement of lamella sheets towards outer AF tissue (Figure 2E). AF tissue sections stained with picrosirius red and observed under polarized light microscopy confirmed a clear transition between outer and inner AF (Figure 2F). Outer AF appeared orange/red, adjusting to a more green/yellow color within the transition zone, before being almost invisible in the inner region, which demonstrated the transition between inner AF and NP. 

### 3.2. Mimicking the AF Tissue

**Figure 3:**
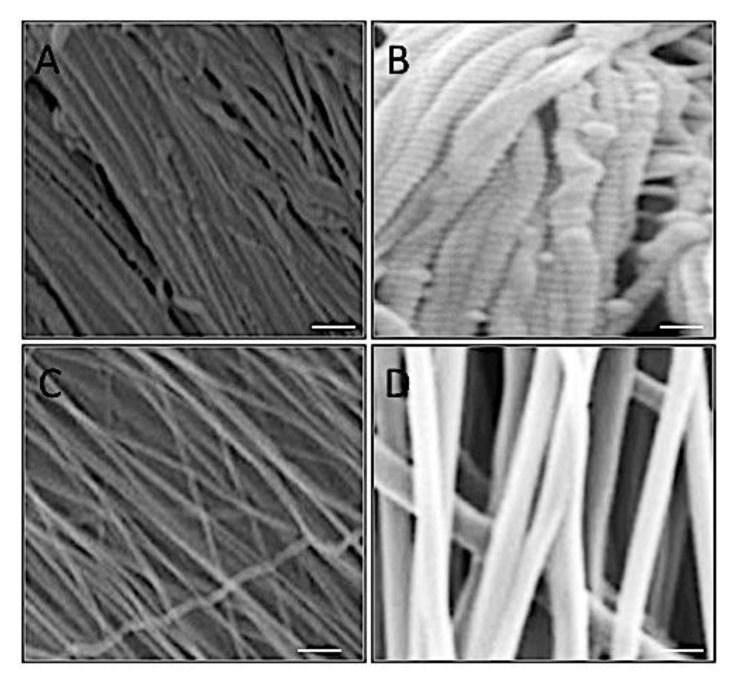
Scanning Electron Microscopy images demonstrating collagen fibers (**A**,**B**) compared to electrospun polycaprolactone fibers (**C**,**D**) at ×1000 (A and C) and ×6000 (B and D) magnification. Scale bar, A and C = 50 μm; B and D = 5 μm).

The structure and morphology of AF collagen fibers were examined and compared to fabricated electrospun PCL fibers. The native tissue demonstrated a densely packed, organized fibrous structure (Figure 3A,B). Electrospun fiber scaffolds presented a similar arrangement of uniaxial fibers suggesting architectural mimicry of the native tissue (Figure 3C,D). However, at higher magnification differences in fiber morphology were observed, with collagen fibers displaying characteristic D-banding pattern and roughened topography (Figure 3B), compared to smooth even surfaces for the electrospun fibers (Figure 3D).

### 3.3. Material Characterization of AF Electrospun Biomimics

#### 3.3.1. Fiber Properties

**Figure 4:**
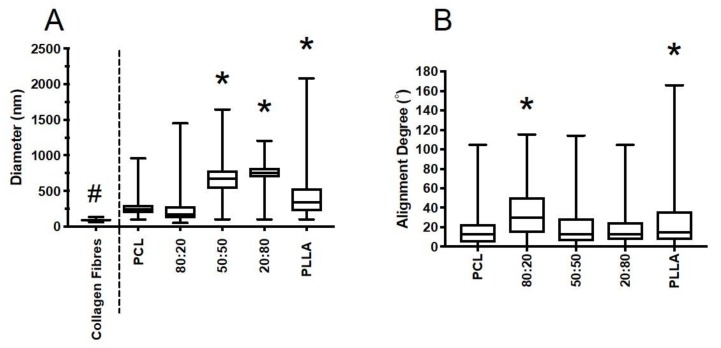
Box and whisker plots representing (**A**) fiber diameter for collagen fibers and polymer groups, and (**B**) fiber alignment for electrospun PCL, PLLA and their blends (PCL:PLLA). Statistical comparison performed with Kruskal-Wallis with Dunn’s multiple comparison test (*p* < 0.05; # all groups significantly different compared to collagen fiber diameter; * represents significant difference compared to PCL fibers).

Fiber diameter for outer AF collagen and electrospun scaffolds were measured and compared (Figure 4A). Collagen fiber diameters were within a relatively tight interquartile range (IQR) (63–136 nm, median 92.8 nm) and were significantly finer than the synthetic electrospun fibers fabricated. Collagen fibers were approximately one third the size of PCL-PLLA 80:20 fibers, which were the finest out of all electrospun fibers fabricated (IQR 99–960 nm, median 242 nm). Overall, the majority of fiber diameters for all electrospun scaffolds fabricated were within the submicron range (<1 μm). Comparison of 100% PCL and 100% PLLA demonstrated PLLA to yield larger diameter fibers with the greatest spread (median 357 nm, IQR 111–2084 nm), whereas PCL fibers were finer (median 234 nm, IQR 99–960 nm). Addition of PLLA to PCL resulted in fiber diameter increasing with rising PLLA content, where 50:50 and 20:80 blends were approximately double the thickness compared to 100% PCL.

As presented in Figure 4B, the majority of PCL and PLLA fibers were aligned within 20°, with median values being 17° (PCL) and 25° (PLLA), respectively. Addition of PLLA to PCL had no obvious effect on fiber alignment. Greatest change was observed for 80:20, with median fiber alignment being 38° off the vertical axis. As a by-product of the electrospinning process, all polymers had a number of fibers oriented almost perpendicular to the main direction of rotation, thus slightly skewing the data for the bulk of fibers collected [10,11]. 

#### 3.3.2. DSC

Thermal properties of all electrospun scaffolds were determined (Table 1). As expected, the melting temperature for PLLA was considerably higher than PCL for 100% pure samples, and this remained relatively unchanged for blended scaffolds. PCL crystallinity was slightly less than PLLA; however, an overall reduction in total crystallinity percentage was obtained for all blended samples compared to 100% pure samples (*p* < 0.05). Results revealed a gradual decrease in total percentage crystallinity for PCL and PLLA blends, which was the lowest for 50:50.

#### 3.3.3. Tensile Testing

**Figure 5:**
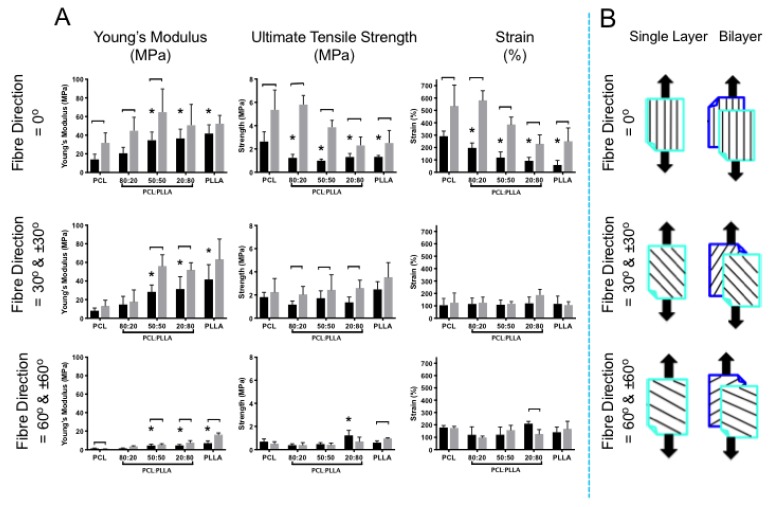
(**A**): Tensile properties for aligned electrospun fiber scaffolds for PCL, PLLA and their blends (PCL:PLLA). Bi-layered scaffolds (grey bar) were compared to single layers (black bar) to determine the effect of opposing lamella structure when under tensile load. Statistical comparison performed with two-way ANOVA and Bonferroni post-tests (single-bilayer comparison) Tukey’s post-test (single layer comparison to PCL) (*p* < 0.05; *n* = 8). Stars (*) represent statistical significance compared to single layer PCL. Down-caps line represents statistical significance between single vs. bilayer of the same blend. (**B**): Schematic: diagrammatic legend illustrating single and bi-layered scaffolds pulled to failure with fiber orientation 0°, 30°, and 60° relative to the direction of load. Black arrows indicate load direction.

Tensile properties for single layer scaffolds and bilayer scaffolds of PCL, PLLA, and their blends were measured by applying a force parallel to the main longitudinal axis of fibers (Figure 5). As expected for single layer, tensile properties were generally weaker for fibers not directly aligned to the direction of testing. Greatest stiffness, strength and strain were obtained for fibers preferentially aligned (0°) to the direction of tensile force. For 100% PCL scaffolds, increasing the angle between the main fiber orientation and direction of load had a negative impact on Young’s modulus and strength, with stiffness decreasing by 43% for 30° and 90% for 60°, and ultimate tensile strength decreasing by 31% for 30° and 73% for 60°. A sudden decrease in strain was also observed when 100% PCL fibers were no longer aligned preferentially to the load direction, though this did not demonstrate a linear relationship, with elongation reducing by 64% and 39% for 30° and 60°, respectively. 

Overall, the addition of PLLA had a considerable effect on tensile properties. This was particularly noticeable for aligned fibers (0°), where scaffolds became significantly stiffer but less elastic as PLLA content increased, and tensile strength similarly decreased with inclusion of PLLA. Similar, yet less striking trends were observed for fibers oriented at 30° and 60°, where the greatest impact of PLLA inclusion was reflected in the Young’s modulus data, with scaffolds becoming stiffer as PLLA content increased. 

Marked changes in tensile properties for 0° and ±30° bilayer scaffolds were observed for all polymer combinations investigated. A double layer resulted in stiffer scaffolds, which followed a near-identical trend with increasing PLLA content. Ultimate tensile strength was generally higher for bilayer, but notable increases in maximum strength were only observed for 0° scaffolds, where bilayers were 51% stronger than single layer for 100% PCL. Similarly, bilayers oriented at 0° resulted in noticeable increases in strain, demonstrating 46% more elongation than single layer for 100% PCL. Ultimate tensile strength barely increased when bilayer scaffolds were tested at ±30° and ±60° (100% PCL), though significant increases were observed for ±30° (20:80, 50:50, and 80:20) and ±60° for 100% PLLA. Elongation almost doubled for all polymers when 0° bilayer scaffolds were pulled to failure, yet there was no obvious change in maximum strain for ±30° and ±60°. 

#### 3.3.4. WCA

**Figure 6:**
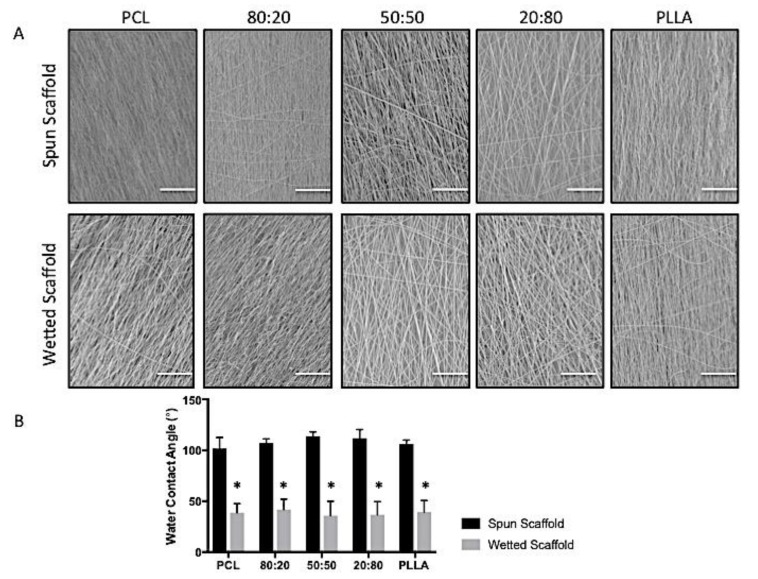
(**A**) SEM micrographs of electrospun fibers for PCL, PLLA and blends (PCL:PLLA) as-spun and after wetting in culture media (dried) (Scale bar = 100 μm). (**B**) Water Contact Angles for electrospun fibers (PCL, PLLA and blends (PCL:PLLA)) as-spun and dried following treatment with cell culture media. Statistical comparison performed with two-way ANOVA and Bonferroni post-tests (* *p* < 0.05; *n* = 5).

The surface hydrophilicity of all scaffolds was investigated by measuring the WCA on as-spun scaffolds and scaffolds wetted in cell culture media and dried prior to testing. As shown from the SEM images in Figure 6A, this media treatment had no effect on fiber or bulk scaffold topographies for all polymer groups investigated. Figure 6B presents WCA measurements before and after wetting. The results showed no significant difference across the spun formulations (test group) neither before nor after the treatment. Irrespective of test group, the WCAs were significantly reduced following media treatment, with apparent transition from hydrophobic (all groups > 100°) to more hydrophilic surfaces (all groups < 40°). 

### 3.4. In Vitro Cell Response to AF Electrospun Biomimics

**Figure 7:**
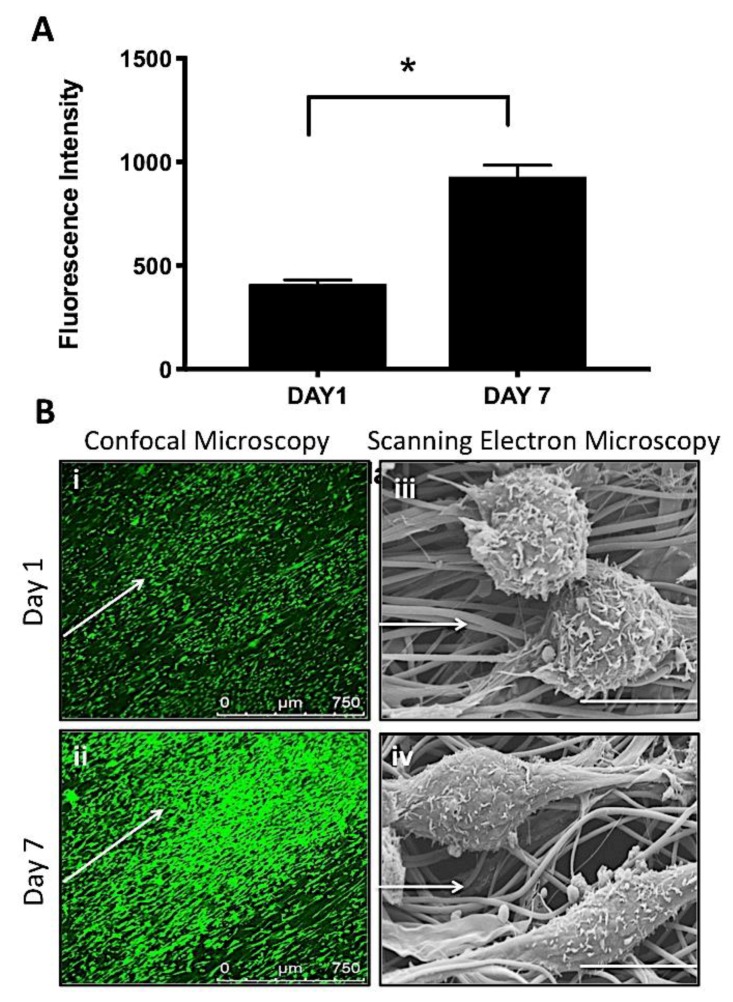
In vitro response of AF cells on PCL/PLLA 50:50 electrospun scaffolds. (**A**) Demonstrates metabolic activity of AF cells at 1 and 7 days using AlamarBlue assay (data reported as mean ±SD and statistical comparison performed with Unpaired t-test and Welch’s correction test (* *p* < 0.05; *n* = 3). (**B**) Confocal microscopy images (i and ii, scale bar = 750 μm) and SEM micrographs (iii and iv, scale bar = 10 μm) of AF cells after 1 and 7 days culture. White arrows indicate main fiber direction.

Based on findings from the material characterization of PCL, PLLA and their blends, Alamar blue was used to quantitatively evaluate AF cell metabolic activity after one and seven days in culture (Figure 7a) on PCL:PLLA 50:50 only. Over this period, a significant increase (two-fold) was observed suggesting cells were viable and metabolically active. Low magnification confocal images (Figure 7b (i and ii)) of phalloidin stained cells clearly demonstrate an increase in cell number over the seven days in culture and their orientation parallel to the underlying aligned fibers. This was further apparent from high magnification SEM images over time, where cells exhibited a shift from rounded to more elongated morphologies that were aligned in the direction of the underlying fibers after seven days in culture (Figure 7b (iii and iv)). 

## 4. Discussion

Due to a paucity of intact normal human IVD tissue being available for study, researchers often use porcine IVD tissue due to its anatomical and biomechanical similarities to human [12,13,14,15]. Thus, for the purposes of replicating AF tissue, a morphological analysis of porcine AF tissue was undertaken to better define its architecture. SEM and histology observations (Figure 2) demonstrated a highly organized structure consisting of two distinct regions: outer and inner AF tissue. In contrast to inner AF, the lamellae in outer AF were tightly packed, stacked layers with fibers precisely aligned parallel to each other. Gamori staining presented collagenous materials in blue regardless of type or structure, with observable change in color intensity between outer and inner AF. This difference may be attributed to variances in collagen type, density, and/or fiber arrangement. In addition, the grades of birefringence colors under polarizing microscopy following picrosirius red staining would suggest organized collagen to be concentrated mainly in the outer region (red to yellow), while less organized fibers could be solely present in the inner region (green to yellow) [16,17]. The absence of birefringence in the central NP region reflects the hydrated, proteoglycan-rich nature of this tissue, which lacks the collagen content and organization observed within the AF. While the persistence of notochordal cells with the porcine NP may produce a more gelatinous matrix than that seen in adult human tissue, the similarities in AF architecture and IVD biomechanics to that previously observed in healthy human AF [12,15] suggested that porcine tissue was an acceptable model for design of electrospun fibrous scaffolds.

Electrospinning was utilized to develop fibrous scaffolds with controlled fiber diameter and orientation that mimicked the individual AF lamellae. As the AF’s unique function is directly reliant on the hierarchical ultrastructure of its collagen fibrils, we compared native AF collagen fibers to electrospun polyester (Figure 3). There were notable similarities between native collagen and electrospun fibers when comparing SEM images, which demonstrated comparable densely packed, organized fibrous structures. However, collagen fibers were considerably thinner than all electrospun polyester groups (Figure 4a) with collagen fibers presenting an average diameter of approximately 90 nm, which is inline with values published for similar tissues of other species [18] whereas electrospun synthetic fibers presented broad fiber distributions, which is also typical of their behavior [18,19,20,21,22,23]. Two synthetic biopolymers and their blends were successfully electrospun to create aligned fibrous scaffolds. Inclusion of PLLA to PCL increased the fiber diameter with blends 50:50 and 20:80 being approximately twice the thickness compared with 100% PCL. This change in fiber diameter for blended scaffolds could be due to the higher inherent viscosity of PLLA compared to PCL, where inherent viscosity is dependent on monomer unit size [24,25]. However, there remains sufficient overlap in the range of diameters between collagen and polyester fibers to continue application of these synthetic materials, as they should still confer mechanical and biological properties sufficient to restore AF tissue function. 

Despite optimizing the mandrel’s speed of rotation, collection of well-aligned fibers was difficult due to the bending instability of the polymer jet [26]. However, results indicate the majority of fibers were relatively well aligned to the direction of rotation for all polymer solutions electrospun, with median values falling below 30° (Figure 4b). Oriented fibers are intended to mimic the fiber alignment of native AF, and hence support adhered cells to secrete organized matrix [27,28]. Bosworth et al. demonstrated 2D aligned sheets and 3D bundles created from aligned PCL electrospun fibers guided tendon fibroblast cells and extracellular matrix to lie parallel to the main fiber direction [29,30]. Hence our aligned fibers from PCL, PLLA, and their blends should provide the topographical cues necessary to support parallel cell growth. 

Differential scanning calorimetry enabled thermal properties and percentage crystallinities for all scaffolds to be determined (Table 1). Thermal properties for the electrospun PCL and PLLA were similar to those previously described [31]. Both PCL and PLLA are semi-crystalline biopolymers, yet electrospinning of these materials resulted in a slight reduction in their overall crystallinity, where rapid solvent evaporation and a large surface area is believed to hinder crystal formation [32,33]. Interestingly, blended solutions yielded a further reduction in total crystallinity (80:20—32%, 50:50—29%, and 20:80—29%). PLLA is known to crystalize more slowly than PCL and coupled with a larger molecular chain could restrict chain mobility [34]. For biodegradable polymers, percentage crystallinity is an important property that affects the rate of degradation. Amorphous regions degrade faster than crystalline; hence our blended scaffolds are likely to degrade more quickly than long-term degrading PCL and PLLA alone [35,36]. The rate of degradation would need to be determined and compared to the rate of AF tissue regeneration to ensure a smooth transition from material to new tissue (e.g. weight-bearing).

Results from mechanical testing of single layer scaffolds suggest that tensile properties are highly dependent on both polymeric blend and fiber direction. The data clearly demonstrate linear relationships for Young’s modulus (increases), strength (decreases), and strain percentages (decreases) for PCL with increasing PLLA content, which was most apparent for fibers oriented parallel to the direction of applied load (Figure 5). These observations were not unexpected, however, as PLLA is known to be a rigid yet brittle material [4], which makes it an unsuitable choice as a sole material for soft tissue applications, such as AF [1,5]. Yet, by combining with PCL—a weak and elastic material [37]—we anticipated positive tensile properties suitable for AF tissue engineering could be elucidated. At 0°, 50:50 and 20:80 PCL to PLLA content resulted in significant increases in scaffold stiffness compared to 80:20, which further highlights the impact PLLA has in this polymer blend. The modulus of single lamella sheets of human AF range from 59–136 MPa [38] and comparison to these current data revealed 0° 50:50 and 20:80 blends to be 41% and 38% weaker than the lowest reported AF lamella. Furthermore, comparison to human AF lamella strength (3.6–10.0 MPa) [38], demonstrates all polymer groups tested to failure were considerably lower, where 0° PCL (yielded highest strength) was 27% weaker than the lowest measure of human AF strength. However, we anticipate the inclusion and integration of cells and their secreted matrix will lead to an increase in stiffness and strength over time [5].

Similar trends were observed for 30° and 60° (single layer scaffolds), however, modulus and strength for 60° oriented fibers were notably reduced. Decreases in tensile properties, irrespective of material content, were expected for single layer where fibers were predominantly oriented at 30° or 60° to the direction of loading as these parallel fibers (unaligned to tensile direction) easily separate from each other when force is applied. This is because less force is transferred along the length of the fiber and a lack of inter-fiber bonding reduces the fibers’ resistance to the load, whereas fibers oriented parallel to the direction of load will transmit this force down the length of the polymeric chains within each fiber [39]. Furthermore, fibers oriented at wider angles relative to the load direction (60°) will be easier to separate as even fewer fibers will cross-over this plane or be drawn to change orientation and resist the applied tensile force. Fiber separation may contribute to material nonlinearity and anisotropy in both aligned electrospun scaffolds and native AF, where application of a flexional force caused collagen fibrils to separate in bovine intervertebral discs [40,41,42,43]. Our findings are in agreement with Holzapfel et al. [44] where strong anisotropic behavior of single AF lamella was observed following tensile loading; and Nerurkar et al [45], who revealed anisotropic mechanical behavior for single layer PCL electrospun fibers when their scaffolds were similarly tested at multiple directions (0°, 15°, 30°, 45°, and 90°) to the applied load. Impact on strain was less affected by changing fiber orientation with minimal differences in maximum strain observed for fibers held at 30° and 60° to the direction of loading.

Similar to single layer scaffolds, trends in mechanical behavior were observed for bilayers with changing PLLA content and fiber direction against load. In addition, and as anticipated, testing of dual fiber sheets yielded significant increases in tensile properties, which may be attributed to friction between the two layers causing raised shear stresses that need to be overcome whilst undergoing tensile loading [45,46]. This was most apparent in modulus and strength for 50:50 and 20:80 bilayer with ±0° and ±30° oriented fibers. Significant increases in strain percentages were also evident for ±0° 20:80 and 50:50 bilayers compared to single layer scaffolds. Besides highlighting the need to incorporate PLLA in order to improve PCL’s inherent poor stiffness, these data demonstrate the importance of multi-layer construction and determining their impact on essential mechanical properties. The tensile data of bilayers demonstrated ratios of 50:50 and 20:80 where fibers oriented ±30° yielded the greatest moduli (56 and 52 MPa), strengths (2.4 and 2.6 MPa), and maintained strains (117 and 187%), suggesting their improved functionality under load. Literature reports the tensile modulus of human AF lamellae to range between 18 and 45 MPa [38]. Comparison to our bilayer scaffolds revealed the modulus to be greater than the highest reported value of human AF by 21% (50:50) and 14% (20:80). 

The surface properties of these polymers and their blends plays a key role in determining their functional application, for example, their hydrophilicity can impact on cellular interactions and adhesion to the scaffold surface. Wetting scaffolds in cell culture media is a simple technique to significantly reduce the hydrophobicity of synthetic biopolymers, such as PCL and PLLA [47,48]. As shown in the SEM images (Figure 6a), and as expected, this technique has no effect on surface morphology as it relies on proteins within the media adsorbing onto the scaffold surface and providing the binding groups for cells to attach [48]. Consequently, water contact angle of electrospun scaffolds was substantially reduced for all groups following submersion in media for 12 h (Figure 6b). 

Many studies have demonstrated electrospun PCL:PLLA blended scaffolds to be biocompatible and supportive of appropriate phenotype for a wide variety of cell types, including bovine AF cells [7,24,49,50,51]. Accordingly, a short-term culture study of bovine AF cells on electrospun fibers was undertaken to ensure this type of scaffold did not have any detrimental effect on cell response. Electrospun PCL:PLLA 50:50 scaffolds were selected as these demonstrated the most appropriate material properties and lowest content of PLLA, making it more cost effective to manufacture; PCL:PLLA 20:80 exhibited similar characteristics, but PLLA is a more expensive polymer, which could have a significant impact in the future manufacturing costs and overall purchase cost of the device. As shown in Figure 7a, AF cells were metabolically active over the seven-day period investigated and this activity increased with time. As there was a noticeable increase in cell number after seven days culture (Figure 7b), this rise in metabolic activity can be attributed to cell proliferation.

It should also be noted that the uniform orientation of electrospun fibers influenced cell alignment and morphology. The confocal and SEM images demonstrated a clear change in cell morphology after 7 days, where cells transitioned from rounded to elongated and morphologies spread parallel to the underlying fibers in response to the fibers’ contact guidance cues (Figure 7b). Given the fusiform shape of AF cells within outer annulus lamellae are extended along the collagen fiber direction [52], the use of aligned scaffolds is particularly advantageous to ensure appropriate cell morphology and production of oriented collagen matrix [53].

## 5. Conclusions

This study aimed to identify the ideal polymeric material combination to create electrospun fibrous lamellae that closely mimic the morphological and mechanical properties of the AF. We determined electrospun fibers made from 50:50 and 20:80 blends of PCL and PLLA demonstrated ideal mimicry of the natural AF and yielded tensile properties within the range of human AF. Furthermore, selection of 50:50 blends (due to lower material costs) exhibited optimal structural integrity and supported desirable cellular response in vitro, suggesting this scaffold is a promising biomaterial for application in herniated intervertebral discs that require AF tissue repair and regeneration. 

## Figures and Tables

**Table 1:** Thermal properties (Melting temperature, Tm; Enthalpy of Fusion, ΔH_f_) and crystallinity percentages for electrospun polymeric scaffolds: polycaprolactone (PCL), poly(L-lactic) acid (PLLA), and their blends.

PCL				PLLA			Total Crystallinity (%)
% Ratio PCL:PLLA	Tm (°C)	ΔH_f_ (J/g)	Crystallinity (%)	Tm (°C)	ΔH_f_ (J/g)	Crystallinity (%)	
100:0	56	50.67	36.32	-	-	-	36.32
80:20	55.23	35.81	26.71	160.2	5.98	6.43	32.10
50:50	56.24	19.2	13.76	164.6	13.9	14.93	28.69
20:80	55.33	8.63	6.42	163.2	27.28	22.87	29.29
0:100	-	-	-	161.1	36.22	38.95	38.95
